# Evaluation of cytotoxicity and genotoxicity of some Philippine medicinal plants

**DOI:** 10.4103/0973-1296.80683

**Published:** 2011

**Authors:** Christine Chichioco-Hernandez, Jakub Wudarski, Lieven Gevaert, Luc Verschaeve

**Affiliations:** *Bioorganic and Natural Products Laboratory, Institute of Chemistry, College of Science, University of the Philippines, Diliman, Quezon City 1101, Belgium*; 1 Gentaur BVBA, Kampenhout, Belgium; 2 Laboratory of Toxicology, O.D. Public Health and Surveillance, Scientific Institute of Public Health, Brussels, Belgium

**Keywords:** Cytotoxicity, genotoxicity, medicinal plants, plant extracts, Pogostemon cablin, Ricinus communis

## Abstract

The genotoxicity and toxicity of ethnomedicinal Philippine plants, which include *Cassia fistula, Derris elliptica, Ficus elastica, Gliciridia sepium, Michelia alba, Morus alba, Pogostemon cablin* and Ricinus communis, were tested using the Vitotox assay. The plants are used traditionally to treat several disorders like diabetes, weakness, menorrhagia, headache, toothache and rheumatism. The dried leaves were homogenized for overnight soaking in methanol at room temperature. The resulting alcoholic extracts were filtered and concentrated in vacuo and tested for their genotoxicity and cytotoxicity using Vitotox®. Results showed that the medicinal plants that were tested are not genotoxic nor cytotoxic, except for *R. communis* and *P. cablin*, which showed toxicity at high doses (low dilutions) in the absence of S9.

## INTRODUCTION

According to the World Health Organization, 80% of the population in Asian and African countries rely on traditional medicine as their main source of health care. The interest in herbal products worldwide as a re-emerging health aid is fueled by the rising costs of drugs. However, there is limited scientific evidence regarding the safety of the plant sources. The efficacy and safety of herbal products depend on the safety of its sources, and this should be established before these are developed as herbal medicinal products.

As part of our ongoing effort to establish the safety of herbal products, several methanolic extracts were prepared from several Philippine plants used as traditional medicine. These plants are used for various conditions like diabetes, weakness, menorrhagia, headache, toothache and rheumatism.[1] Different fractions of *Derris elliptica* exhibited a broad-spectrum antimicrobial activity.[2] Various rotenoids were isolated from its roots.[3,5] There are no reports regarding the biological activity of *Ficus elastica. Morus alba* is used as a traditional medicine in several countries like Italy and Tunisia,[6] Jordan,[7] Korea[8] and India.[9] Its extracts exhibited various bioactivities like antivenom,[10] inhibition of nitric oxide and prostaglandin E2 production in peritoneal macrophages,[11] stimulation of skeletal muscle 5’-AMP-activated protein kinase,[12] *in vitro* antileukaemic activity,[13] antiobesity,[14,15] antidiabetic,[16,17] hepatoprotective and free radical-scavenging activities,[18] antiinflammatory[19] and antidopaminergic.[20] An antibacterial agent from its root is active against oral pathogens.[21] Its root bark also contains a glycoprotein used as a component of antidiabetic therapy in oriental medicine.[22] The leaf extract of *Ricinus communis* was found to be cytotoxic in several human cancer lines,[23] and also exhibited an antidiabetic action.[24] *Gliciridia sepium* is used as a folkloric medicine in Columbia[25] and is used in Guatemala to treat protozoal infections.[26] It is used as an antifeedant[27] and contains stigmastanol glucoside[28] and saponins.[29] It shows an antidermatophyte activity[30] *Cassia fistula* exhibits antibacterial, antifungal,[31] hepatoprotective,[32,33] antitumor,[34] larvicidal and ovicidal activities against filarial and malarial mosquitos.[35] *Pogostemon cablin* contains anti-trypanosomal sesquiterpenes hydroperoxides[36] and a cytotoxic chalcone.[37]

In this paper, we describe the results of a genotoxicity and cytotoxicity evaluation of these plant extracts using the bacterial Vitotox^®^ test.

## MATERIALS AND METHODS

### Plant material

*D. elliptica, F. elastica, Morus alba, R. communis, G. sepium, C. fistula, P. cablin and Michelia alba* leaves were collected from the University of the Philippines, Diliman Campus, and submitted to Dr. Jose Vera Santos Herbarium, Institute of Biology, University of the Philippines, Diliman, for authentication. Voucher specimens were also deposited.

### Plant extraction

Approximately 100 g of dried leaves were homogenized and then soaked overnight in 100% methanol (MeOH). The resulting MeOH extract was filtered and concentrated *in vacuo* using a rotary evaporator (Heidolph: Starenstraße 23 D-93309 Kelheim, Germany).

### Genotoxicity and cytotoxicity assay

The plant extracts were tested using Vitotox assay as described previously.[38] Briefly, the test employs two different bacterial reporter strain constructs of *Salmonella typhymurium* (TA 104) based on an SOS-response. One has a luciferase gene under the control of the recN promoter, which leads to light production when DNA is damaged (TA 104-recN2-4 strain or Genox strain) while the second one contains *lux* -genes under the control of a constitutive promoter so that the light production is not influenced by genotoxic compounds (pr1 or Cytox strain). It serves as an internal control wherein, if the light production goes up, the test compounds affect the *lux* gene in a different way than damaging the DNA. On the other hand, a decrease in light production would indicate a toxic response.

Light emission was recorded after the addition of the plant extracts to the bacteria every 5 min during 4 h using a luminometer. The plant extracts were tested with and without the presence of metabolizing S9 fraction. The signal to noise ratio (S/N) or, specifically, the light production of exposed bacteria divided by the light production of non-exposed bacteria, is automatically calculated for each measurement. S/N is calculated for both strains separately as well as the ratio between the maximum S/N values of the exposed over the control strain. A substance is considered genotoxic when:

-max S/N (genox)/ max S/N (cytox) >1.5

-max S/N in genox shows a good dose-effect relationship

-max S/N (genox/cytox) shows a good dose-effect relationship

The extracts are not considered genotoxic if S/N (genox) increases rapidly within the first 30 min or immediately shows a high value as SOS induction is not yet possible within this short period of time.

## RESULTS AND DISCUSSION

*D. elliptica, F. elastica, M. alba, R. communis, G. sepium, C. fistula, P. cablin and M. alba* MeOH were evaluated using the Vitotox assay. The plant extracts were dissolved in dimethylsulfoxide to give a concentration of 1 mg/mL, and a dilution series was prepared from 1/1 to 1/128 in the sample preparation step.

The final dilutions of the samples in the measurement plate are 1/100 to 1/12,800 compared with the original stock solution of 1 mg/mL. 4-Nitroquinoline oxide (4-NQO) was used as a positive control without S9 metabolic activation while benzopyrene (Bαp) was used as the positive control requiring S9 metabolic activation. The final concentrations of 4-NQO and Bαp in the measurement plate are 4 pbb and 8 ppm, respectively.

**Figure 1:**
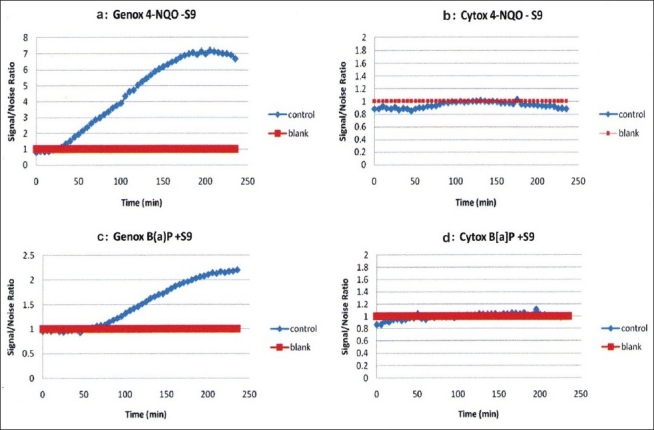
Genotoxicity and cytotoxicity using positive controls; 4-nitroquinoline as positive control without metabolic activation as shown in panels a and b and benzopyrene as positive control requiring metabolic activation as shown in panels c and d

**Figure 2:**
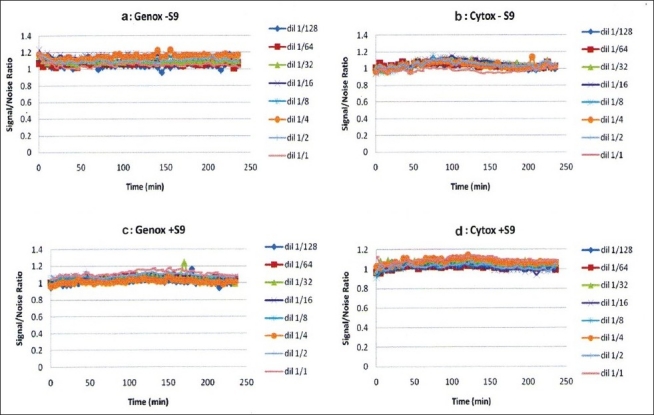
Genotoxicity and cytotoxicity profile of *Cassia fistula*. Similar profiles were obtained from the extracts of *Derris elliptica, Ficus elastica, Michelia alba, Gliciridia sepium* and *Morus alba*

**Figure 3:**
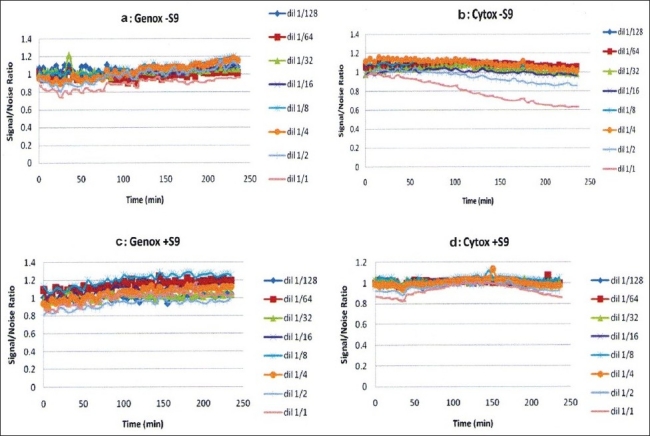
Vitotox^®^ profile of the *Ricinus communis* plant extract

**Figure 4:**
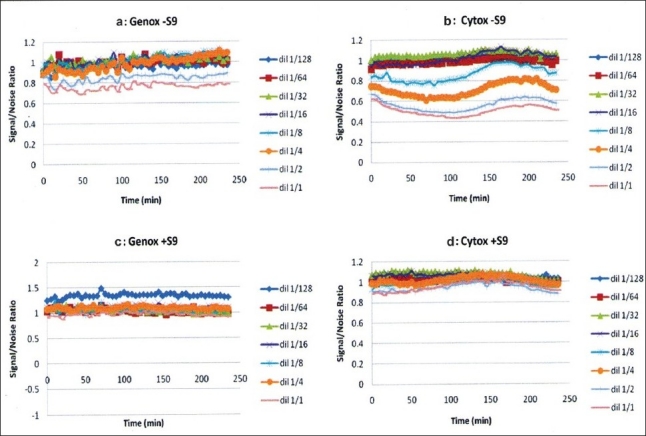
Vitotox^®^ profile of the *Pogostemon cablin* plant extract

4-NQO was found to be genotoxic by inducing light production over an S/N of 1.5, but was not cytotoxic, as shown in Figure 1a and b. On the other hand, Bαp was similarly found to be genotoxic with metabolic activation as seen in Figure 1c and d. *D. elliptica, F. elastica, M. alba, G. sepium, C. fistula* and *M. alba* MeOH displayed non-genotoxicity and non-cytotoxicity behavior in all concentrations tested, as shown in Figure 2. However, *R. communis* and *P. cablin* were found to be cytotoxic at high concentrations. *R. communis*, which was previously evaluated as cytotoxic in several human cancer cell lines,[23] was found to be cytotoxic without metabolic activation at a concentration of 10 ppm and also showed a concentration-correlation effect with its cytotoxic behavior, as shown in Figure 3. *P. cablin*, which contains a cytotoxic chalcone,[37] was also found to be cytotoxic without metabolic activation at higher concentrations, as shown in Figure 4. None of the plant extracts tested was found to be genotoxic.

To the best of our knowledge, this is the first report of the evaluation of the genotoxic and cytotoxic effects of the tested medicinal plant extracts using the Vitotox^®^ assay. Our results are rather reassuring about the safety of most of the assayed plant extracts, but should be confirmed by further in-depth investigations.
